# Neural differences in self-perception during illness and after weight-recovery in anorexia nervosa

**DOI:** 10.1093/scan/nsw092

**Published:** 2016-06-27

**Authors:** Carrie J. McAdams, Haekyung Jeon-Slaughter, Siobahn Evans, Terry Lohrenz, P. Read Montague, Daniel C. Krawczyk

**Affiliations:** ^1^Department of Psychiatry, University of Texas at Southwestern Medical School, Dallas, TX 75390, USA; ^2^Psychiatry, Texas Health Presbyterian Hospital of Dallas, Dallas, TX 75321, USA; ^3^Virginia Tech Carilion Research Institute, Virginia Tech, Roanoke, VA 24016, USA; ^4^Computational Psychiatry Unit, Virginia Tech, Roanoke, VA 24016, USA; ^5^Wellcome Trust Centre for Neuroimaging, University College London, 12 Queen Square, London, WCIN 3BG, UK; ^6^Department of Physics, Virginia Tech, Blacksburg, VA 24061, USA,; ^7^Center for Brain Health, University of Texas at Dallas, Dallas, TX 75390, USA

**Keywords:** eating disorders, medial prefrontal cortex, self-reflection, fMRI, psychiatry

## Abstract

Anorexia nervosa (AN) is a severe mental illness characterized by problems with self-perception. Whole-brain neural activations in healthy women, women with AN and women in long-term weight recovery following AN were compared using two functional magnetic resonance imaging tasks probing different aspects of self-perception. The Social Identity-V2 task involved consideration about oneself and others using socially descriptive adjectives. Both the ill and weight-recovered women with AN engaged medial prefrontal cortex less than healthy women for self-relevant cognitions, a potential biological trait difference. Weight-recovered women also activated the inferior frontal gyri and dorsal anterior cingulate more for direct self-evaluations than for reflected self-evaluations, unlike both other groups, suggesting that recovery may include compensatory neural changes related to social perspectives. The Faces task compared viewing oneself to a stranger. Participants with AN showed elevated activity in the bilateral fusiform gyri for self-images, unlike the weight-recovered and healthy women, suggesting cognitive distortions about physical appearance are a state rather than trait problem in this disease. Because both ill and recovered women showed neural differences related to social self-perception, but only recovered women differed when considering social perspectives, these neurocognitive targets may be particularly important for treatment.

## Introduction

Anorexia nervosa (AN) is a serious psychiatric illness associated both with an inability to maintain one’s body weight, and altered perceptions about one’s body shape and weight (American Psychiatric Association, 1994). Problems in the internal construct of self, including both social and physical aspects of one’s identity, have been proposed to be a core feature of this illness leading to food restriction (Stein and Corte, 2007; Fairchild and Cooper, 2010). A failure to develop a secure sense of identity might promote disordered eating behaviors in many ways. Acceptance of one’s actual body shape and size, a difficult problem in AN (Skrzypek *et al.*, 2001), requires an internal sense of physical self. Identifying why one’s emotions might be distressing requires understanding of one’s emotions. However, starvation appears to reduce distressing emotions in AN, a factor that may exacerbate the disease (Brockmeyer *et al.*, 2011), and alexithymia, the ability to identify emotions, is elevated in AN (Beadle *et al.*, 2013). Women with AN also show high needs for external sources of approval (Amianto *et al.*, 2011); that approval might be sought by seeking out socially-imposed beliefs such as the thin ideal. Problems in making decisions (Guillaume *et al.*, 2015), and navigating interpersonal relationships (Treasure and Schmidt, 2013) are also observed in AN; both behaviors require self-reflection to identify how pros and cons of a situation relate to oneself. Fundamentally, decisions related to self—emotions and values—are ubiquitous, and might contribute to many characteristics and traits that lead to both the restricted food intake and preoccupations about body image that characterize AN.

Currently, there is a limited understanding of the pathology of AN, and even less understanding of how pathology and treatments are related (Dalle Grave, 2011). Although weight-restoration is essential for recovery from AN, more than half of adult patients relapse after treatment (Steinhausen, 2002; Keel and Brown, 2010). Therapies that consider body-image, identity, and self-esteem are typically utilized after weight-restoration but the specific cognitive, physiological, and emotional changes that result in sustained recovery have not been clearly identified (Murphy *et al.*, 2010; Lazaro *et al.*, 2011; Brockmeyer *et al.*, 2013; Watson and Bulik, 2013; Hay *et al.*, 2014; Kelly *et al.*, 2014). Utilization of functional magnetic resonance imaging (fMRI) tasks to characterize specific neurocognitive problems in self-perception in AN may improve our ability to understand the disease and design targeted treatments.

Neurobiologically, medial prefrontal cortex (MPFC), posterior cingulate cortex and the temporoparietal junctions have been closely related to self-perception and evaluation in healthy populations (Denny *et al.*, 2012). Differences in neural activations during social and self-perception in these regions have been reported in AN (Muhlau *et al.*, 2007; McCormick *et al.*, 2008; Miyake *et al.*, 2010; McAdams and Krawczyk, 2011, 2014; Miyake *et al.*, 2012; Schulte-Ruther *et al.*, 2012; McFadden *et al.*, 2014). In this study, we were interested in determining whether women with and recovered from AN showed similar neural activations during social and physical self-perception. The study goal was to assess whether neural differences related to self-perception might provide a better understanding of both the pathogenesis of the disease and the process of recovery from AN.

We compared neural activations to self-stimuli in three groups of subjects: healthy comparison women (HC), women with AN and women in long-term weight-recovery following AN. Using these groups, we evaluated whether neural differences in processing self-stimuli could be associated with either the development of the disease (present in both ill and recovered but not healthy women) or stage of the disease (present in ill or recovered but not both patient groups). We adapted the social appraisal task from our prior study (McAdams and Krawczyk, 2014), hypothesizing that women with and recovered from AN would show frontal lobe differences during social self-evaluations. We utilized a self and stranger face-viewing task to probe neural activity during physical self-reflection (Kishida *et al.*, 2012), hypothesizing that self-image would more strongly engage visual regions during the illness than after recovery.

## Methods

### Participants

Subjects provided written informed consent to participate, approved by the UT Southwestern Institutional Review Board. A total of 59 women (age range 20–45 years) participated: 19 HC, 22 women currently with AN (AN-C, DSM IV criteria within prior 6 months) and 18 women with sustained weight recovery (AN-WR, 24 months with BMI > 19.0). Subjects were interviewed using the Structured Clinical Interview for DSM-IV to confirm course and history of AN for AN-C and AN-WR groups, and absence of eating disorders (EDs) in the HC group. More clinical details about the groups are provided in the Supplemental Methods S1.

Subjects completed a structured interview for current depression (Quick Inventory of Depressive Symptoms, QIDS-CR), and anxiety (Structured Interview Guide for the Hamilton Anxiety Scale, SIGH-A). ED symptoms were assessed with the Eating Disorder Examination Questionnaire (EDE-Q) in the AN-C and AN-WR group; all subjects also completed the Eating Attitudes Test (EAT). The Wechsler Abbreviated Scale of Intelligence (WASI) provided an estimate of intelligence.

### Neuroimaging tasks

The Social Identity-V2 task consisted of three different conditions: Self (e.g. ‘I believe I am deceitful’), Friend (‘I believe “my friend” is moody’) and Reflected (‘“My friend” believes I am proud’), personalized with the name of a female friend. Each statement was presented above the terms Agree and Disagree for 4 s, and a response was selected each trial, followed by a jittered fixation period of 4, 6 or 8 s. There were 48 statements in each of three runs; 16 statements for each condition resulting in a total duration of 8 minutes per run. All statements for each condition were sequential, and condition order pseudorandomized across runs. The Social Identity-V2 task differed from the original Social Identity task (McAdams and Krawczyk, 2014) (See Supplemental Methods S2). During each trial, each subject indicated a rating of agreement using a hand-held button, yielding behavioral data as a reaction time and response (Agree or Disagree).

For the Faces task, each subject viewed 15 images of their own face (F-Self) and 15 images of a stranger (F-Other), each presented for 4 s with a jittered time-interval (Kishida *et al.*, 2012). The stranger image matched the subject as closely as possible in age, skin-tone, and hair color; all images differed but included both eyes open, a neutral or positive expression, with a variety of head tilts. One image appeared every 8–30 s, with a fixation cross between images. Because of technical difficulties, four women in the AN-C group did not complete the Faces task.

### Statistical analyses of clinical and behavioral data

Demographic, clinical and behavioral comparisons were completed in SPSS v.21 (SPSS, Inc., Chicago). Measures obtained for all groups (Age, BMI, WASI, QIDS, SIGH-A, EAT) were compared using analysis of variance (ANOVA) with post-hoc comparisons, using a Bonferroni corrected *P* < 0.05. Measures collected only from the patient groups (EDE-Q, age of onset) were compared with a two-sample *t*-test (*P* < 0.05). Mean reaction times and mean percentage of trials showing agreement in the Social Identity-V2 task were compared across groups and conditions using multivariate ANOVA followed by Bonferroni post-hoc comparisons (criterion of *P* < 0.05).

### MRI acquisition and analysis

Images were acquired with a 3T Philips Achieva MRI scanner, using a 1-shot gradient T2*-weighted echoplanar image sequence sensitive to blood oxygen level-dependent (BOLD) contrast with a repetition time (TR) of 2 s. For Social Identity-V2, the echo time (TE) was 35 ms, and the flip angle was 0°; volumes were composed of 36 axial slices (4-mm thick, no gap). For the TE was 30 ms, and a flip angle was 90°; volumes were composed of 34 axial slices (4-mm thick, no gap). For both tasks, each slice was acquired with a 22.0 cm^2^ field of view, a matrix size of 64 × 64 and a voxel size of 3.4 × 3.4 × 4 mm. Functional images were acquired during 4 runs (3 for ‘Social Identity-V2’, each 480 s; 1 for Faces, between 540 and 600 s). High-resolution MP-RAGE 3D T1-weighted images were acquired for anatomical localization with the following imaging parameters: TR = 2100 ms, TE = 3.7 ms; slice thickness of 1 mm with no gap, a 12° flip angle, and 1 mm^3^ voxels.

Preprocessing consisted of spatial realignment to the first volume of acquisition, normalization to the MNI standard template, and spatial smoothing with a 6 mm 3D Gaussian kernel. The voxel time-series was high pass filtered (128 s). fMRI task data were analyzed using Statistical Parametric Mapping software (SPM8, Wellcome Department of Imaging Neuroscience London, www.fil.ion.ucl.ac.uk/spm) run in Matlab 2012 (http://www.mathworks.com) and viewed in both SPM and xjview (http://www.alivelearn.net/xjview8/). An event-related design extracted the BOLD signal during the 4-s presentation of each statement or image and a general linear model (GLM) created contrast images of each event (events: Social Identity-V2: Self-Agree, Self-Disagree, Friend-Agree, Friend-Disagree, Reflected-Agree, Reflected-Disagree; Faces: F-Self, F-Other). Evoked activation was assessed using multiple regression analysis set as boxcar functions. Each regressor was convolved with a canonical hemodynamic response function provided in SPM8 and entered into the modified GLM. Parameter estimates (e.g. β values) were extracted from this GLM analysis for each regressor. A first level analysis evaluated task contrasts (Social Identity-V2, three contrasts*:* Self–Friend, Self-Agree–Self-Disagree, Reflected–Self; Faces task, one contrast: F-Self – F-Other). Supplementary analyses were also completed for the Social Identity-V2 task, considering valence of the adjectives rather than behavioral responses (Supplemental Analysis S1).

To identify group differences, a whole-brain ANOVA followed by pairwise comparisons identified regions of interest (ROI) (threshold: cluster-corrected *P*_fwe_< 0.05 with voxel-wise *P* < 0.005, uncorrected). Age was a covariate of no interest for the whole-brain analyses. For each ROI showing significant group differences from the exploratory whole-brain comparisons, parameter estimates (β values) were extracted for each subject using Marsbar (Brett *et al.*, 2002). The parameter estimates were exported to SPSS, and the groups compared using an ANOVA followed by pairwise comparisons (Bonferroni-corrected *P* < 0.05).

Two methods considered whether clinical measures were related to the neural activations in the tasks. Pearson’s correlations considered whether clinical symptoms (QIDS-CR, SIGH-A, EDE-Q) correlated with the extracted parameter estimates within the seven ROIs obtained from the whole-brain ANOVAs. These correlations were performed across all patient–participants (AN-C and AN-WR), and separately for each patient group [corrected threshold: *P*-value of 0.007 (*P* = 0.05/7 ROIs)]. Second, whole-brain regressions explored whether the first-level images for each of the four contrasts correlated with the clinical symptoms for the patient-participants only (cluster *P*_FWE_ < 0.05, with voxel-wise *P* < 0.005). Psychoactive medication effects were considered with a group × medication multivariate ANOVA using extracted parameter estimates for patient-subjects [criterion: *P*-value of 0.007 (*P* = 0.05/7 ROIs)].

## Results

### Demographic and clinical comparisons

Clinical and demographic differences are provided in Table 1. The AN-C group had a lower body mass index than both the HC and AN-WR groups. All three groups differed on the overall EAT, and its Dieting and Bulimia Subscales. The AN-C and AN-WR groups differed from the HC group, but not each other, for the EAT Oral Subscale, QIDS-CR and SIGH-A. The AN-C group had more symptoms on the EDE-Q and all subscales compared to the AN-WR group. In sum, all significant psychiatric differences in the groups were related to ED symptoms, not anxiety or depression symptoms.

**Table 1.** Participant characteristics

	HC (*n* = 19)		AN-C (*n* = 22)		AN-WR (*n* = 18)		*F_2,56_*	*P*
	Mean	SD	Mean	SD	Mean	SD		
Age (years)	27.9	6.0	27.6	7.6	29.6	8.1	0.74	0.483
ED Onset Age (years)			17.4	6.0	14.0	3.7	2.11^d^	0.041
Intelligence quotient (WASI)	122.53	9.0	117.8	8.3	118.5	13.8	1.16	0.322
Current body mass index	22.5	2.4	17.6	1.5	22.8	2.7	35.23	<0.001^a^^,^^c^
EAT	3.3	3.9	39.0	17.9	15.7	9.9	43.62	<0.001^a^^,^^b^^,^ ^c^
EAT-Dieting	1.7	2.9	20.4	10.9	10.1	7.2	28.35	<0.001^a^^,^^b^^,^ ^c^
EAT-Bulimia	0.4	0.8	9.5	4.4	3.2	2.1	52.24	<0.001^a^^,^^b^^,^ ^c^
EAT-Oral	1.3	1.6	8.1	5.9	2.0	2.0	19.44	<0.001^a^^,^^b^
Quick Inventory of Depression	1.6	2.1	6.7	5.3	5.1	4.4	7.75	0.001^a^^,^^b^
Hamilton anxiety	2.3	2.8	10.3	8.7	8.3	6.6	7.80	0.001^a^^,^^b^
EDE-Q								
EDE-Q–global			3.94	1.44	2.14	1.36	4.03^d^	<0.001
EDE-Q–restraint			4.06	1.48	1.81	1.41	4.87^d^	<0.001
EDE-Q–eating concern			3.17	1.85	1.29	1.01	3.78^d^	0.001
EDE-Q–shape concern			4.60	1.58	2.68	1.64	3.75^d^	<0.001
EDE-Q–weight concern			3.92	1.41	2.78	1.85	2.22^d^	0.032

HC, healthy comparison women; AN-C, women with anorexia nervosa; AN-WR, women in weight recovery after anorexia nervosa. ED, eating disorder; EAT, Eating Attitude Test; EDE-Q, Eating Disorder Examination Questionnaire.

*Post hoc* statistical comparisons were performed using Bonferroni correction:

^a^AN-C differ significantly from HC (*P* < 0.05).

^b^AN-WR differ significantly from HC (*P* < 0.05).

^c^AN-C differ significantly from AN-WR (*P* < 0.05).

^d^t-value for the comparison of AN-C and AN-WR (df = 38, *P* < 0.05).

### The Social Identity-V2 task

***Behavioral data.*** Response (Agree or Disagree) and reaction time for each condition (Self, Friend and Reflected) were obtained each trial (Supplementary Table S1). There were no group or condition differences in reaction times, indicating that all groups spent similar amounts of time evaluating each statement. The AN-C group showed less agreement across all conditions compared with both the AN-WR and HC groups [percent agree, AN-C = 52%; AN-WR = 56%, HC = 55%; F(2,56)= 7.094, *P* = 0.001]. All groups agreed more during ‘Self’ than for ‘Friend’ and ‘Reflected’ [percent agree, Self = 57%, Friend = 52%, Reflected = 53%; *F*(2,56)= 11.847, *P* < 0.001].

**Fig. 1.**
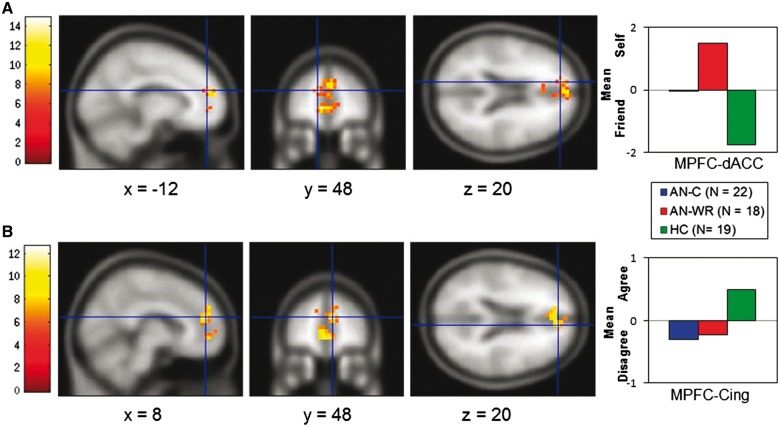
The top row **(A)** shows the MPFC-dACC, centered on its peak MNI coordinates, from the main effect of group ANOVA for the Self–Friend contrast of the Social Identity-V2 task, with the parameter estimates (β values) extracted from this ROI, averaged for each group, on the right. The bottom row **(B)** shows the MPFC-Cing, centered on its peak MNI coordinates, from the main effect of group ANOVA for the Self-Agree–Self-Disagree contrast of the Social Identity-V2 task with its parameter estimates, averaged for each group and plotted on the right.

***Social self-perception: self–friend.*** The Self–Friend contrast directly compares activation of neural regions for self-evaluations with friend-evaluations. Across all groups, direct evaluation of oneself activated regions in the bilateral parietal cortex, whereas evaluations of one’s friend activated MPFC and the posterior cingulate/precuneus (Supplementary Figure S1). Considering each group separately, the HC group had more clusters for Friend than Self; the AN-C group showed no neural differences; and the AN-WR group showed more activations for Self than Friend (Supplementary Table S2). A region in the MPFC extending into the dorsal anterior cingulate (MPFC-dACC) differed in the whole-brain ANOVA comparing all groups (Figure 1A, Table 2). This area was more active during Self trials for the AN-WR group and more active during Friend trials for the HC group, with little modulation in the AN-C group. Similarly in pairwise comparisons, both the AN-WR–HC and AN-C–HC comparisons had more neural activity for Self than Friend and no differences were observed in the AN-C and AN-WR comparisons (Supplementary Table S3).

**Table 2.** Characteristics of all ROI from the whole-brain comparisons and regressions^a^

Condition and region	Neural ROI characteristics						β values from ROI mean (SD)			Group comparisons using β-values		
	Volume (mm^3^)	Cluster size	Peak Z	MNI			AN-C	AN-WR	HC	*F_2,56_*	*P*	Effect sizes
				x	Y	z						
*Social Identity-V2: Self–Friend*												
MPFC-dACC	10 718	232	4.34	−12	48	20	−0.03 (0.9)	1.11(1.1)	−1.3(1.1)	24.9	<0.001^b^^,c,d^	0.54^b^, 0.74^c^, 0.50^d^
*Social Identity-V2: Self-Agree–Self-Disagree*												
MPFC-Cing	6283	136	4.01	8	48	20	−0.30 (0.4)	−0.22(0.4)	0.50(0.6)	14.3	<0.001^b^^,c^	0.58^b^, 0.56^d^
*Social Identity-V2: Reflected–Self*												
LIFG/Insula	2356	51	3.95	−56	8	4	−0.08 (1.0)	−0.89(0.8)	1.08(1.0)	18.0	<0.001^b^^,c,d^	0.49^b^, 0.73^c^, 0.40^d^
RIFG/Insula	3650	79	4.29	36	32	8	0.07 (0.7)	−1.21(0.7)	0.64(1.1)	23.1	<0.001^c^^,d^	0.71^c^, 0.70^d^
dACC	3603	78	4.07	4	32	32	0.05 (1.1)	−2.08(1.8)	0.05(1.2)	14.4	<0.001^c^^,d^	0.58^c^, 0.58^d^
*Faces: F-Self–F-Other*												
LFusi/temporal	5960	129	3.78	−34	−60	−14	0.56 (0.5)	−0.16(0.4)	0.03(0.5)	10.5^e^	<0.001^b^^,d^	0.47^b^, 0.62^d^
RFusi /temporal	4712	102	3.57	34	−76	−10	0.62 (0.4)	−0.22(0.7)	−0.06(0.7)	9.2^e^	<0.001^b^^,d^	0.50^b^, 0.60^d^
*EDE-Q Global Regression for Patient–Participants Only using Social Identity-V2: Reflected–Self Contrast*												
dACC-EDEQ	2495	54	4.52	4	32	40	0.04 (1.0)	−1.49(1.4)	0.06(1.5)	8.8	<0. 001^c^^,d^	0.53^c^, 0.47^d^
LMFG	3280	71	4.13	−36	28	36	0.27 (1.1)	−0.83(1.1)	0.68(1.2)	9.0	<0. 001^c^^,d^	0.44^c^, 0.54^d^
RMFG	1894	41	4.36	48	20	40	0.43 (1.1)	−0.90(0.9)	0.13(1.5)	6.6	0.003^c^^,d^	0.55^c^, 0.38^d^

^a^All Clusters at *P*_*FWE*_ < 0.05. The volume of each voxel is 46.2 mm^3^. Peak Z Score is at the MNI coordinates for the specified anatomical location. HC, healthy comparison women, AN-C, currently-ill women with anorexia, and AN-WR, weight-recovered women with anorexia. MPFC, medial prefrontal cortex, dACC, dorsal anterior cingulate, Cing, cingulate, LIFG, left inferior frontal gyrus, RIFG, right inferior frontal gyrus, LFusi, left fusiform, RFusi, right fusiform, LMFG, left middle frontal gyrus; RMFG, right middle frontal gyrus. Effect sizes computed from Cohen's d using means of extracted b values.

^b^AN-C differ significantly from HC (*P* < 0.05).

^c^AN-WR differ significantly from HC (*P* < 0.05).

^d^AN-C differ significantly from AN-WR (*P* < 0.05).

^e^df for the Faces Task was 2, 52.

***Social Self-Relevance: Self-Agree–Self-Disagree.*** The Self-Agree–Self-Disagree contrast isolates neural activations related to self-relevance of the stimuli (Supplementary Table S4). Group differences in the MPFC extending into the cingulate were observed, with the extracted parameter estimates showing that the HC utilized this area more for Self-Agree trials but both the AN-C and AN-WR activated it more for Self-Disagree trials (Table 2, Figure 1B; Supplementary Table S5). These results suggest that women with AN, both during illness and after sustained recovery, use MPFC-Cing more for cognitive processes related to self-disagreement whereas healthy women use this area more for cognitive processes related to self-agreement.

**Fig. 2.**
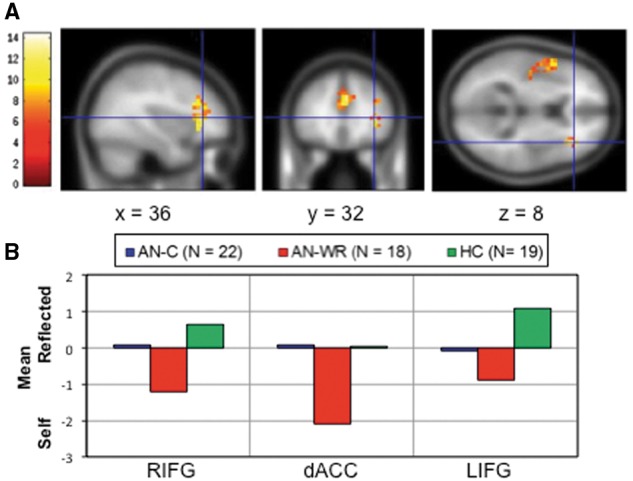
Three clusters were identified in the main effect of group ANOVA for the Reflected–Self contrast of the Social Identity-V2 Task. The upper row **(A)** is centered on the peak response in the RIFG, with the dACC cluster also visible in the middle panel and the LIFG cluster, as it extends into the insula, also visible in the far right panel. In the bottom row **(B)**, parameter estimates for the Reflected–Self contrast were extracted for each ROI, and the average for each group is shown.

***Social Evaluation: Reflected–Self.***The Reflected–Self contrast typically leads to the recruitment of additional social cognitive neural regions (D’Argembeau *et al.*, 2007), and this was observed in the all subjects group map (Supplementary Table S6 and Supplementary Figure S2). In the group comparison, the bilateral inferior frontal gyri extending into the anterior insulae [L Inferior Frontal Gyrus (LIFG), R Inferior Frontal Gyrus (RIFG)], and the dACC differed for all three groups (Table 2, Figure 2). Consideration of the β values from these regions shows that the AN-WR group engaged all regions more for Self than for Reflected evaluations (negative values in Figure 2B for Reflected-Self contrast), and differed from the HC and AN-C group in all regions. The AN-C group overall showed relatively little differentiation in the Reflected–Self contrast (values near zero), and differed from the HC group only in the LIFG. Pairwise comparisons supported these results with differences in the HC–AN-C contrast, the HC–AN-WR contrast and in the AN-C–AN-WR contrasts (Supplementary Table S7).

### The Faces task

**Fig. 3.**
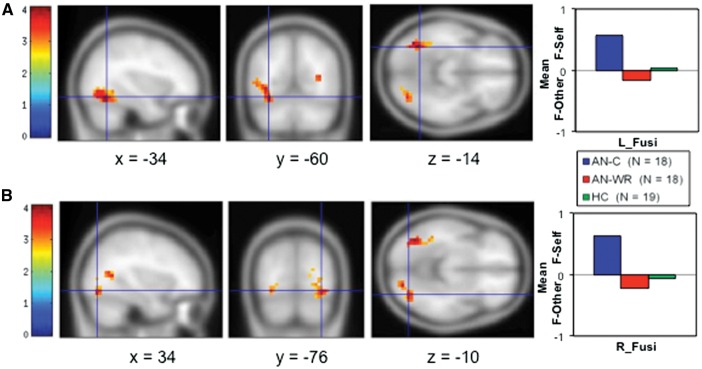
Two clusters were identified in comparison of the AN-C–AN-WR groups in the F-Self–F-Other contrast of the Faces task. In the top row **(A)**, a cluster in the LFusi extending into the temporal lobe showed more activation in the AN-C group for F-Self images than in both the AN-WR and HC groups. In the bottom row **(B)**, a cluster in the right fusiform (RFusi) extending into the temporal lobe also showed more activation in the AN-C group for F-Self images than in both the AN-WR and HC groups.

In the Faces task, subjects passively viewed their own image (F-Self) or a comparable picture of a stranger (F-Other). Across all subjects and in the AN-WR and HC groups, presentation of one’s own image led to stronger activations of the middle cingulate, bilateral insula and inferior frontal gyri whereas viewing the stranger’s image activated the bilateral temporoparietal junctions and precuneus (Supplementary Table S8 and Supplementary Figure S3). The AN-C group did not show any regions with elevated activity for the F-Other trials, and instead had clusters in the temporal and parietal regions in the F-Self trials. Whole-brain differences were observed only in the pairwise comparison of the AN-C and AN-WR groups (Table 2, Figure 3). One cluster was in the left middle temporal gyrus, extending into the fusiform gyrus (LFusi), and the other was in the right middle temporal gyrus, extending into the fusiform gyrus (RFusi). In both cases, the extracted parameter estimates from these ROIs confirmed more F-Self activity in the AN-C group than in both the HC and AN-WR groups (Figure 3).

### Clinical symptoms, medications, and neural activations

Parameter estimates were extracted from the seven ROIs identified in the whole-brain group comparisons and correlated with the clinical symptom scales (EDE-Q, SIGH-A, QIDS). Consistent with the whole-brain results, several of the regions that differed for the AN-C and AN-WR groups were also correlated with the EDE-Q-Global (RIFG, *r* = 0.488, *P* = 0.001; dACC, *r* = 0.441, *P* = 0.004; LFusi, *r* = 0.587, *P* < 0.001; RFusi, *r* = 0.474, *P* = 0.003). However, this relationship was not present within any region when each patient group was considered separately. Neither the depression (QIDS) nor anxiety (SIGH-A) scales correlated with any ROIs. Second, because there were 4 contrasts and 3 clinical scales, 12 whole-brain regressions explored whether clinical scales directly correlated with whole-brain responses. Three clusters were identified in the social self-evaluation contrast (Social Identity-V2, Reflected–Self) regression with the EDE-Q global scale (Table 2, Supplemental Figure S4). No clusters were identified in any of the regressions for the other three contrasts or related to the depression and anxiety scales. The group × medication use ANOVA found no significant differences related to medication use (Supplementary Table S9).

## Discussion

We examined how the neural processes engaged during four cognitive contrasts related to self-perception differed amongst women with AN, those with sustained weight-recovery from AN, and healthy women (See Supplemental Patient Perspectives S1). First, in the self-relevance contrast, both ill and weight-recovered women utilized MPFC and the cingulate more when disagreeing about descriptors whereas the HC group utilized these regions when agreeing with the terms. In this area and contrast alone, the AN-C and AN-WR groups were similar to each other but differed consistently from the HC group, suggesting a persistent biological trait difference related to self-relevance. Second, the weight-recovered women showed different neural activations compared to both the ill and the healthy women in several frontal regions during the two conditions related to social self-perception and evaluation. We hypothesized that both groups with AN would show altered engagement of frontal regions during social self-evaluation. That hypothesis was formulated because alterations in the salience network including both the dACC and bilateral insula have been observed in many psychiatric illnesses (Goodkind *et al.*, 2015; Downar *et al.*, 2016). Instead, we found differences in the utilization of these salience network regions during social self-evaluation only for the AN-WR group, suggesting a neural difference that may be important for sustained weight-recovery. Finally, we hypothesized that ill women might show more neural activations in visual regions when viewing themselves compared to others, relative to the other groups, because problems in physical self-perception are associated with acute illness. We confirmed that hypothesis, observing increased bilateral fusiform activations for self-stimuli in the ill but not the other groups.

MPFC was utilized for different cognitive functions by the HC group (more active in Friend and Self-Agree trials) than by both the AN-C and AN-WR groups (more active in Self and Self-Disagree trials). This result suggests that the biological processes that mediate social self-perception and self-relevance may be fundamentally different in AN, indicative of a possible trait related to development of the disease. van Veluw and Chance (2014) conducted a meta-analysis proposing that MPFC and the superior temporal gyri are key neural regions involved in self-recognition. Oberndorfer *et al.* (2011) reported reduced ability to modulate MPFC in response to task difficulty amongst weight-recovered women with AN, supporting the idea that dysfunction of MPFC may contribute to development of this disease. Another neural region that has been proposed as a trait related to pathology in AN is the ventral striatum (Frank, 2015). Intriguingly, in healthy men and women, the connectivity of MPFC to the ventral striatum has been associated with trait self-esteem, whereas functional activation of the MPFC for self-relevance correlated with state self-esteem (Chavez and Heatherton, 2015). Self-esteem has been related to ED severity, social problem-solving and outcomes (Paterson *et al.*, 2007; Karpowicz *et al.*, 2009). In concert, these data suggest that dysfunction in corticostriatal circuits contribute to both symptoms of disordered eating as well as the alterations in self-perception observed in AN. Without a strong sense of reward when experiencing either positive social or physiological (food) feedback, women with AN may both lack both a secure sense of self-identity and motivation to eat. Future work to connect self-esteem, corticostriatal circuitry and illness trajectory in AN may lead to a mechanistic model of how neurobiological factors contribute to the development of AN.

One of the study goals was to determine if regional neural responses related to self-stimuli differed for the AN-C and AN-WR groups because understanding how changes related to self-perception are related to clinical course may help with treatment recommendations and targets. These differences could result in normalization of a brain region that was previously dysfunctional during illness (AN-WR and HC same, AN-C differs) or changes in brain regions only amongst the sustained weight-recovered group (AN-WR differs, HC and AN-C same). Both types of results were observed in this study.

In the physical self-perception task, we found that the AN-C group had increased processing of self-images compared with stranger-images in visual regions relative to the HC and AN-WR groups, suggesting that sustained-weight recovery normalizes neural responses for physical self-perception. Consistent with these results, Inoue *et al.* (2015) utilized near-infrared spectroscopy to examine right temporal lobe responses to self and stranger faces in adolescents with AN, finding increased blood flow to these regions for face stimuli. In sum, these data suggest both that hyperactivation of early visual regions for self-stimuli may alter physical self-perception during acute disease and that biological aspects of physical self-perception may be resolved after sustained weight-recovery. This is encouraging, as it suggests physical distortions related to body-image are transient rather than persistent traits that predispose to AN, or that treatments currently utilized for AN succeed, resolving the body-image components in this disease.

Alternatively, in the social self-evaluation contrast of the Social Identity-V2 task, we observed differences in activations in three frontal regions, the bilateral anterior insula and dACC, for the AN-WR group relative to both other groups. The dACC and anterior insula are both parts of the salience network, and have been proposed to serve as a switch related to motivation and environment that is disrupted in mental illness (Downar *et al.*, 2016). Changes in utilization of the salience network for self-stimuli may be important for sustained weight-recovery following AN. Importantly, there were no behavioral differences for the Social Identity-V2 task in the AN-WR and HC comparisons. This suggests that neural activations can be more sensitive in detecting internal cognitive differences than behavioral measures.

Because this was a cross-sectional rather than longitudinal study, differences in the AN-WR group relative to the other groups could reflect neural compensations, pre-existing differences predictive of recovery or a biological trait suppressed during acute disease. We favor the neural compensation hypothesis because outpatient treatment of AN typically includes individual psychotherapy and cognitive therapies that focus on self-perception, body-image, mentalization, and self-esteem (Lazaro *et al.*, 2011; Brockmeyer *et al.*, 2013; Watson and Bulik, 2013; Hay *et al.*, 2014; Kelly *et al.*, 2014), all constructs related to social self-evaluations. One area that differed during reflected third person evaluations compared to direct first person evaluations in the recovered women was the dACC, a region that has been directly related to the difficulty of a task (Shenhav *et al.*, 2014). This suggests that after weight-recovery from AN, self-evaluations may be more challenging, perhaps due to internal conflicts in self-assessments. A recent meta-analysis of the neural correlates related to psychotherapy in depression and anxiety also reported changes in the frontal lobes, cingulate gyrus and precuneus following treatments, and proposed that these neural differences result from changes that these therapeutic interventions create for self-related information processing (Messina *et al.*, 2013). Since we show that sustained weight-recovery includes neural differences related to social self-evaluation, treatment of AN may benefit by targeting this specific neurocognitive process. We also found that frontal regions were related to the EDE-Q global score during social self-evaluation for patient-participants, linking ED symptomatology to this social process. In concert, these data suggest that both decreases in ED symptoms and sustained weight-recovery from AN alter frontal brain activations during social self-perception.

ED clinical symptoms correlated with neural activations in response to self-stimuli for several of the ROIs that differed in the groups. Most commonly, clinical imaging studies include one clinical group and one comparison group, and symptoms in the clinical group are correlated with neural activations. When we combined the patient-groups, we found that neural activations in the dACC, right inferior frontal gyrus and bilateral fusiform gyri correlated with the eating symptoms, confirming the relevance of these regions in the disease process. However, within each group, symptom scales were not correlated with neural activations, underscoring the importance of considering both the course of illness as well as current symptoms during assessments of AN. In this study, considering the course of illness led to more robust neurocognitive differences related to self-perception than measures of acute ED symptoms.

One limitation of Social Identity-V2 task is that valence and behavioral response were confounded, both because participants (across all groups) agreed with few negative stimuli and disagreed with few positive stimuli, and because a much larger set of stimuli would be necessary to separate condition, behavior, and valence (See Supplementary Material Analysis 1). Interestingly, the insula and inferior frontal gyri, areas more activated in both patient groups compared with the controls during direct self-evaluations, were recently associated with negative self-evaluations in healthy subjects (Cabanis *et al.*, 2013). Ambwani *et al.* (2015) recently reported perceptual impairments related to positive stimuli in AN. Previously, we observed reduced neural activations in the precuneus and superior temporal sulcus following positive social interactions in both ill and weight-recovered women with AN (McAdams *et al.*, 2015). Further research to determine whether differences in behavioral responses and neural activations depend upon the valence of self-stimuli is needed.

Another limitation involves the variability of the clinical characteristics of the patient groups. Groups were defined primarily on body mass index during the last 6 months (AN-C) and 2 years (AN-WR). These criteria were selected because maintenance of weight within a healthy range reduces the mortality and morbidity of AN, with most relapses reported during the first 2 years after treatment (Strober *et al.*, 1997). Second, this criterion is more objective than individual patient reports of symptoms. Additionally, because this study focuses on evaluating neural differences related to cognitions about self-perception, examining individuals that are acutely malnourished (i.e. patients with AN immediately upon entry to a treatment program or severely underweight) would be inappropriate, as numerous cognitive problems, such as difficulties with set-shifting and central coherence, are observed following starvation even in non-clinical samples (Pender *et al.*, 2014). The goal of this study was to assess whether neurocognitive differences in self-perception contribute to the pathology of AN, a process best served by focusing on patients after nutritional stabilization, rather than examining the progressive loss of cognition associated with starvation. Nevertheless because on the day of the scan, six AN-C subjects had a normal BMI and six AN-WR subjects reported significant ED symptoms, we excluded those individuals for a *post-hoc* analysis, and repeated the whole-brain ANOVAs, with similar findings (Supplemental Analysis 2). Similarly, although both clinical groups showed moderate and similar amounts of depression and anxiety, neural differences in these self-perception tasks were not related to either depression or anxiety, only the ED symptoms. These results suggest neural changes in social self-evaluation are specifically related to ED symptoms, and may be important in both the development of the disease and its clinical course.

In conclusion, neural regions were connected both with cognitive processes underlying self-perception as well as illness and recovery in AN, leading to new possibilities for neural and cognitive treatment targets. The differences in MPFC during self-relevance suggests a biological risk trait, a problem that might be approached with deep brain stimulation or transcranial magnetic stimulation, techniques just beginning to be explored in AN (Lipsman *et al.*, 2013; McClelland *et al.*, 2015). Most excitingly, neural differences related to engagement of the salience network during social self-evaluation were found in women with sustained weight-recovery compared to both other groups, suggesting that successful long-term weight recovery from AN involves not only reductions in acute symptoms of an ED but also changes in the neurocircuitry engaged for self-stimuli. More research is needed to determine whether interventions that explicitly target social self-evaluation can improve outcomes from AN.

## Supplementary data

Supplementary data are available at *SCAN* online.

Supplementary Data
