# Supplementary material for: Neural differences in self-perception during illness and after weight-recovery in anorexia nervosa
Source: Soc Cogn Affect Neurosci. 2016 Jun 27;11(11):1823–31. doi: 10.1093/scan/nsw092 (PMC5091684; doi:10.1093/scan/nsw092)
Supplement: Supplementary Data [file supp_11_11_1823__index.html]

Neural differences in self-perception during illness and after weight-recovery in anorexia nervosa — Neural differences in self-perception during illness and after weight-recovery in anorexia nervosa — Supplementary Data 

# Neural differences in self-perception during illness and after weight-recovery in anorexia nervosa

## Supplementary Data

files

- Supplementary Data - rtf file
