# Supplementary material for: Neural differences in self-perception during illness and after weight-recovery in anorexia nervosa
Source: Soc Cogn Affect Neurosci. 2016 Jun 27;11(11):1823–31. doi: 10.1093/scan/nsw092 (PMC5091684; doi:10.1093/scan/nsw092)
Supplement: Supplementary Data [file supp_nsw092_scan-16-152-File005.rtf]

Supplementary Online Content

McAdams CJ, Slaughter HJ, Evans S, Lohrenz T, Montague PR, Krawczyk, DC. Neural differences in self-perception during illness and after weight-recovery in anorexia nervosa 
Methods S1. Clinical Characteristics of Participants
Methods S2. Differences in Social Identity-V2 Task and previous Social Identity Task.
Table S1: Behavioral Data from the Social Identity-V2 Task
Table S2. Clusters identified in Self - Friend contrast of Social Identity-V2 Task
Table S3. Pairwise differences in Self – Friend contrast of Social Identity-V2
Table S4. Clusters identified in Self-Agree – Self-Disagree of Social Identity-V2 Task
Table S5. Pairwise differences in Self-Agree – Self-Disagree of Social Identity-V2 Task 
Table S6. Clusters identified in Reflected - Self contrast in Social Identity-V2 Task
Table S7. Pairwise differences for Reflected - Self contrast of Social Identity-V2 Task 
Table S8. Clusters identified in F-Self – F-Other contrast of the Faces task
Table S9. Current antidepressant use and parameter estimates for ROIs with Group Effects
Figure S1. Social Identity-V2 task activations for all subjects: Self vs. Friend contrast
Figure S2. Social Identity-V2 task activations for all subjects: Reflected vs. Self contrast
Figure S3. Faces task activations for all subjects:  F-Self vs. F-Other contrast
Figure S4. Regions obtained from regression of the EDE-Q against the patient-participants; as well as the comparison maps for the Reflected-Self main effect of group.
Patient Perspectives S1. Patient interpretations of the task.
Supplemental Analysis S1: Valence Effects
Supplemental Analysis S2: Weight and ED Symptom Effects


Methods S1. Clinical Characteristics of Participants

All subjects in the AN-C group met DSM-IV criteria for anorexia nervosa within the previous 6 months. Most of these subjects (15/22) were scanned during or within three months of completing an intensive eating disorder program (resulting in a few of those patients having a normalized weight at the scan (6 of those 15 with BMI > 18.5 on day of scan); the others were in outpatient treatment, but had also maintained a stable weight, albeit below normal (BMI < 18.0) for at least two months prior to the scan. These criteria were selected to minimize effects of acute weight loss on brain function, but to still allow examination of patients in the process of obtaining treatment for this disease. 
All subjects in the AN-WR group had met DSM-IV criteria for anorexia nervosa previously in their life but had maintained a BMI greater than 19.0, for at least two years, and had resumed menstrual cycles. None of the subjects in the AN-WR group had been treated in an inpatient, partial hospital or residential eating disorder program for at least two years. These subjects were recruited through on-campus flyers, referrals from outpatient clinical providers, and postings on eating disorder advocacy and support sites. 
Prior diagnoses of anxiety disorders or depressive disorders were permissible, but subjects with current major depression, current or past substance dependence, current or past bipolar disorders, and current or past psychotic disorders were excluded. None of the subjects in either the AN-C or the AN-WR groups had met the DSM IV criteria for bulimia nervosa for at least 12 months, although six subjects in each clinical group had met DSM IV criteria for bulimia nervosa in their lifetime. Subjects with lifetime diagnoses of bipolar disorders, psychotic disorders, and substance dependence were excluded, as were subjects with substance abuse during the prior 12 months. None of the subjects had met criteria for a major depressive episode in the month prior to the scan, but a few subjects had met criteria for recurrent major depressive disorder in their lifetime (4 AN-C, 5 AN-WR). Some subjects had met criteria for an anxiety disorder prior to the development of anorexia nervosa (anxiety not otherwise specified (3 AN-C, 3 AN-WR) and generalized anxiety disorder (3 AN-C, 3 AN-WR). Participants on antidepressants whose dosage had not changed for at least 1 month prior to their MRI scans were included (0 HC; 12 AN-C; 7 AN-WR). Comorbidities, and the use of antidepressants, are common amongst adult patients with anorexia nervosa (See Table S9). 


Methods S2. Differences in Social Identity-V2 Task and previous Social Identity Task1
There were three differences in the Social Identity-V2 task compared to the previously published Social Identity task (1). First, a minor change converted the task to three eight minute runs rather than four six minute runs.  Second, the specific adjectives were altered. Many words were of strong valence in the first study (such that everyone agreed (eg, kind, thoughtful) or everyone disagreed (mean, vulgar), and we found that subjects tended to respond most quickly to the most valenced terms, providing less time to acquire data about internal decision-making processes. Additionally, some words were less specific to chronic personality traits than temporary experiences (clean, happy). As such, we created a list of more neutral terms focusing on social interactions, experiences, and personality-trait descriptors. Third, the response options were changed. The latter two changes were related: in the original task, adjectives included many strongly positive or negative terms, intending to examine differences related to agreeing and disagreeing with positive and negative stimuli. However, as nearly all subjects disagreed with negative words and agreed with positive words, and agreement always corresponded to right-hand buttons and disagreement to left-hand buttons, analysis of the cognitive processes associated with agreeing were overwhelmed by the more-substantial hemispheric motor response difference. Hence, we increased the specificity for social-self cognitions by selecting less-valenced terms, and balanced motor responses by reducing the button-press responses to Agree and Disagree, and pseudo-randomly having responses on different sides of the screen. 

Italicized works in the first list indicate the new terms added; crossed-out terms in the second list indicate the words removed. These changes allowed consideration of behavioral responses.

Word List for Social Identity-V2 Task: clumsy, compulsive, conceited, confident, considerate, critical, curious, cynical, deceitful, decisive, dependable, dishonest, disrespectful, easygoing, friendly, greedy, honest, impractical, independent, insecure, intelligent, interesting, mature, messy, moody, optimistic, ordinary, phony, possessive, practical, proud, quiet, realistic, reliable, reckless, sarcastic, selfish, sensitive, serious, sincere, spiteful, stubborn, timid, tough, truthful, untruthful, warm, wise

Word List for previous Social Identity Task clean, conceited, considerate, cruel, deceitful, dependable, dishonest, disrespectful, earnest, friendly, good-natured, greedy, happy, heartless, honest, honorable, insolent, insulting, intelligent, interesting, kind, loyal, malicious, mature, mean, narrow-minded, obnoxious, open-minded, phony, reliable, rude, selfish, sincere, spiteful, thoughtful, thoughtless, trustworthy, understanding, unkind, unselfish, untrustworthy, untruthful, vulgar, warm, wise

1.	McAdams CJ, Krawczyk DC (2014): Who am I? How do I look? Neural differences in self-identity in anorexia nervosa. Soc Cogn Affect Neurosci. 9:12-21.


Table S1. Behavioral Data from the Social Identity-V2 Task

	AN-C	AN-WR	HC	Analysis of Variance	
Behavioral Measure	Mean (SD)	Range	Mean (SD)	Range	Mean (SD)	Range	Group	Condition	Interaction	
Reaction Time, Self	2191 (253)	1800-2513	2217 (252)	1710-2565	2094 (303)	1598-2725	F = 0.87	F = 1.55	F = 0.286	
Reaction Time, Friend	2062 (278)	1592-2800	2102 (266)	1661-2562	2095 (285)	1655-2637	P = 0.42	P =  0.22	P = 0.89	
Reaction Time, Reflected	2212 (245)	1718-2469	2151(254)	1731-2583	2132 (308)	1712-2755				
Percentage Agree, Self	0.54 (0.08)	0.43-0.73	0.60 (0.06)	0.46-0.71	0.60 (0.05)	0.52-0.69	F = 4.44	F = 10.01	F = 0.24	
Percentage Agree, Friend	0.50 (0.06)	0.37-0.61	0.53 (0.06)	0.45-0.70	0.54 (0.06)	0.43-0.65	P = 0.01b	P < 0.001c	P = 0.92	
Percentage Agree, Reflected	0.51 (0.05)	0.43-0.61	0.53 (0.06)	0.42-0.65	0.54 (0.05)	0.46-0.65				
a AN-C, currently-ill women with anorexia, AN-WR, weight-recovered women with anorexia, HC, healthy comparison women. Group: AN-C, AN-WR, HC. Condition: Self, Friend, Reflected.
b AN-C agreed significantly less often with words than both the HC and AN-WR in the Bonferroni pairwise comparisons (P < 0.05).
c Self differed significantly from Friend and Reflected in the Bonferroni pairwise comparisons (P < 0.05).


Table S2. Clusters identified in Self - Friend contrast of Social Identity-V2 Task
Condition and Region	Self or Friend	Volume
(mm3)	Cluster
Size	Peak
Z Score	x	y	z	
Average Effect of Condition, All Subjectsb	
Posterior Cingulate / Precuneus	Friend	6422	139	6.08	4	-56	20	
Right Parietal	Self	17002	368	5.40	32	-56	36	
Medial Prefrontal Cortex	Friend	6006	130	4.89	0	56	32	
Left Parietal	Self	10949	237	4.7	-24	-64	36	
Left Fusiform	Self	2680	58	3.85	-28	-56	-20	
HC Subjects	
Right Medial Temporal Gyrus	Friend	2541	55	6.02	52	-4	-16	
Medial Prefrontal Cortex	Friend	15292	331	5.69	-4	56	32	
Left Medial Temporal Gyrus	Friend	3419	74	5.34	-56	-4	-16	
Posterior Cingulate / Precuneus	Friend	8131	176	5.18	8	-52	16	
Right Insula	Friend	2541	55	4.66	28	20	-20	
Right Parietal	Self	2772	60	3.44	32	-56	36	
AN-C Subjects: NO CLUSTERS	
AN-WR Subjects	
Cingulate and 
Dorsal Anterior Cingulate	Self	7577	164	4.46	4	36	32	
Right Fusiform / Occipital	Self	2726	59	4.35	44	-76	0	
Right Fusiform	Self	4066	88	4.29	28	-52	-16	
Right Middle Frontal Gyrus	Self	6468	140	4.24	48	28	32	
Right Parietal	Self	7022	152	4.04	28	-60	32	
Left Parietal	Self	8501	184	4.03	-28	-48	36	
a Threshold was individual voxel P < 0.005 and cluster-corrected PFWE < 0.05 (corresponded to 51 voxels for this contrast). Volume of each voxel is 46.2 mm3. Peak Z Score refers to the z-score at the MNI coordinates for the specified anatomical location (x,y,z). HC, healthy comparison women, AN-C, currently-ill women with anorexia, and AN-WR, weight-recovered women with anorexia
b. T-contrast maps were used to create the corresponding figure, Figure S1, of the “Average Effect of Condition, All Subjects” to allow color-based displays of the Self and Friend clusters. Table S2 reports on clusters based on the Z-score criteria. Slight differences may result (T-contrasts are more sensitive but less specific).	


Table S3. Pairwise differences in Self – Friend contrast of Social Identity-V2 Taska 
Condition and Region	Volume
(mm3)	Cluster
Size	Peak
Z Score	x	y	z	
HC – AN-C: NO CLUSTERS	
AN-C – HC	
Medial Prefrontal Gyrus	4112	89	3.76	16	56	16	
HC – AN-WR: NO CLUSTERS	
AN-WR – HC	
Medial Prefrontal Cortex	25780	558	4.76	-12	48	20	
Right Insula and Inferior Frontal Gyrus	9563	207	4.48	40	16	-8	
Cerebellum	10487	227	4.38	0	-52	-28	
Medial Temporal Gyrus	6468	140	4.10	52	-4	-16	
Left Parietal	2356	51	3.60	-16	-48	40	
Right Parietal	3373	73	3.58	16	-52	32	
Left Insula and Inferior Frontal Gyrus	3973	86	3.56	-56	12	8	
Left Superior Temporal Gyrus	3095	67	3.50	-48	-32	4	
AN-C – AN-WR: NO CLUSTERS	
AN-WR – AN-C: NO CLUSTERS	
a Threshold was individual voxel P < 0.005 and cluster-corrected PFWE < 0.05 (corresponded to 51 voxels for this contrast). Volume of each voxel is 46.2 mm3. Peak Z Score refers to the z-score at the MNI coordinates for the specified anatomical location. HC, healthy comparison women, AN-C, currently-ill women with anorexia, and AN-WR, weight-recovered women with anorexia	


Table S4. Clusters identified in Self-Agree – Self-Disagree of Social Identity-V2 Task
Condition and Region	Agree or Disagree	Volume
(mm3)	Cluster
Size	Peak
Z Score	x	y	z	
Average Effect of Condition, All Subjectsb		
Occipital Lobe	Disagree	11458	248	5.50	-8	-80	0	
Right Parietal	Disagree	3003	65	4.75	20	-64	44	
Dorsal Anterior Cingulate	Disagree	5174	112	4.06	-12	8	56	
HC Subjects	
Left Temporoparietal Junction	Agree	3973	86	4.63	-40	-72	28	
Left Frontal Gyrus	Agree	2818	61	4.31	-32	20	48	
Medial Prefrontal Cortex	Agree	3373	73	3.83	-4	52	0	
Precuneus	Agree	3557	77	3.60	-16	-44	32	
AN-C Subjects: NO CLUSTERS	
AN-WR Subjects	
Right Parietal	Disagree	4851	105	4.43	24 	-68	44	
Midbrain	Disagree	2495	54	4.43	12	-16	-12	
Calcarine Lobe	Disagree	7762	168	4.25	-8	-80	4	
Left Middle Temporal Gyrus	Disagree	2911	63	4.17	-52	-40	0	
Left Inferior Frontal Gyrus	Disagree	3881	84	4.06	-40	4	36	
Dorsal Anterior Cingulate	Disagree	3604	78	3.94	-4	12	52	
Left Parietal	Disagree	3188	69	3.73	-24	-60	52	
a Threshold was individual voxel P < 0.005 and cluster-corrected PFWE < 0.05 (corresponded to 50 voxels). Volume of each voxel is 46.2 mm3. Peak Z Score refers to the z-score at MNI coordinates for the specified anatomical location. HC, healthy women, AN-C, currently-ill women with anorexia, and AN-WR, weight-recovered women with anorexia.	


Table S5. Pairwise differences in Self-Agree – Self-Disagree of Social Identity-V2 Taska 
Condition and Region	Volume
(mm3)	Cluster
Size	Peak
Z Score	x	y	z	
HC – AN-C	
Medial Prefrontal Cortex	18480	400	4.42	8	48	20	
Precuneus	3049	66	3.96	-16	0	12	
Left Temporoparietal Junction	2356	51	3.62	-36	-72	28	
AN-C – HC: NO CLUSTERS	
HC – AN-WR	
Left Medial Temporal Gyrus	2818	61	3.93	-56	-40	0	
Midbrain	2864	62	3.89	12	-16	-12	
Left Temporoparietal Junction	6791	147	3.81	-36	-72	28	
Posterior Cingulate	4851	105	3.56	0	-40	28	
AN-WR – HC: NO CLUSTERS	
AN-C – AN-WR	
Right Parietal	3095	67	3.74	24	-52	44	
AN-WR – AN-C: NO CLUSTERS	
a Threshold was individual voxel P < 0.005 and cluster-corrected PFWE < 0.05 (corresponded to 50 voxels). Volume of each voxel is 46.2 mm3. Peak Z Score refers to the z-score at the MNI coordinates for the specified anatomical location. HC, healthy comparison women, AN-C, currently-ill women with anorexia, and AN-WR, weight-recovered women with anorexia	


Table S6. Clusters identified in Reflected - Self contrast in Social Identity-V2 Task
Condition and Region	Reflected
Or Self	Volume
(mm3)	Cluster
Size	Peak
Z Score	x	y	z	
All Subjects	
Right Lingual Gyrus	Reflected	3188	69	5.53	16	-80	0	
Left Lingual Gyrus	Reflected	3049	66	5.52	-12	-88	0	
Right Occipital / Fusiform	Self	4204	91	4.93	4	-72	0	
Left Temporoparietal Junction	Reflected	3465	75	4.91	-52	-56	20	
Cuneus	Self	4481	97	4.45	4	-84	12	
Right Parietal	Self	3465	75	4.43	28	-48	-16	
Precuneus	Reflected	4481	97	4.43	0	-60	32	
HC	
Right Lingual Gyrus	Reflected	2495	54	4.58	12	-80	-8	
Left Lingual Gyrus	Reflected	2633	57	4.57	-16	-84	0	
Left Temporoparietal	Reflected	4851	105	4.41	-52	-56	20	
Left Globus Pallidus	Reflected	2541	55	4.21	-20	0	-12	
AN-C	
Left Lingual Gyrus	Self	2125	46	4.63	-12	-92	0	
AN-WR	
Cingulate / Dorsal Anterior Cingulate	Self	12197	264	4.82	0	32	24	
Right Middle Frontal Gyrus	Self	5082	110	4.18	48	24	28	
Right Occipital	Self	3465	75	4.00	4	-84	12	
Left Middle Frontal Gyrus	Self	2633	57	3.60	-48	16	0	
a Threshold was individual voxel P < 0.005 and cluster-corrected PFWE < 0.05 (corresponded to 42 voxels for this contrast). Volume of each voxel is 46.2 mm3. Peak Z Score refers to the z-score at the MNI coordinates for the specified anatomical location. HC, healthy comparison women, AN-C, currently-ill women with anorexia, and AN-WR, weight-recovered women with anorexia.
b. T-contrast maps were used to create the corresponding figure, Figure S2, of the “Average Effect of Condition, All Subjects” to allow color-based displays of the Reflected and Self clusters. Table S6 reports on clusters based on the Z-score criteria. Slight differences may result (T-contrasts are more sensitive but less specific).	


Table S7. Pairwise differences for Reflected - Self contrast of Social Identity-V2 Taska 
Condition and Region	Volume
(mm3)	Cluster
Size	Peak
Z Score	x	y	z	
HC – AC	
Left Insula / Inferior Frontal Gyrus	3373	73	3.97	-56	-12	16	
Right Insula / Inferior Frontal Gyrus	2218	48	3.93	48	-4	16	
AN-C – HC: NO CLUSTERS	
HC – AN-WR	
Right Insula/ Caudate / Putamen	25918	561	5.32	36	32	8	
Left Insula/ Caudate / Putamen	20605	446	4.64	-16	12	12	
Right Middle Frontal Gyrus	2587	56	4.37	44	0	24	
Left Superior Temporal Gyrus	3788	82	4.03	-52	-56	40	
AN-WR – HC: NO CLUSTERS	
AN-C – AN-WR	
Cingulate / Dorsal Anterior Cingulate	7484	162	4.25	0	32	24	
Right Inferior Frontal Gyrus	6422	139	4.13	36	24	24	
Precuneus	2818	61	3.50	16	-76	40	
AN-WR – AN-C: NO CLUSTERS	
a Threshold was individual voxel P < 0.005 and cluster-corrected PFWE < 0.05 (corresponded to 42 voxels for this contrast). Volume of each voxel is 46.2 mm3. Peak Z Score refers to the z-score at the MNI coordinates for the specified anatomical location. HC, healthy comparison women, AN-C, currently-ill women with anorexia, and AN-WR, weight-recovered women with anorexia	

Table S8. Clusters identified in F-Self – F-Other contrast of the Faces taska 
Condition and Region	Self or Other	Volume
(mm3)	Cluster
Size	Peak
Z Score	x	y	z	
All Subjectsb	
Right Insula and Inferior Frontal Gyrus	Self	33726	730	7.13	38	8	-6	
Right Parietal	Self	20698	448	6.77	62	-24	34	
Left Insula and Inferior Frontal Gyrus	Self	15107	327	6.53	-38	8	-6	
Middle Cingulate	Self	22037	477	6.38	6	28	26	
Precuneus	Other	19404	420	6.10	6	-60	30	
Right Temporoparietal Junction	Other	15523	336	5.82	54	-60	26	
Left Temporoparietal Junction	Other	15569	337	5.47	-46	-60	42	
Left Superior Frontal Gyrus	Other	19357	419	5.08	-46	24	38	
HC 	
Right Insula and Inferior Frontal Gyrus	Self	8455	183	4.48	38	8	6	
Left Temporoparietal Junction	Other	8085	175	4.29	-42	-52	34	
Right Temporoparietal Junction	Other	4851	105	4.22	50	-60	30	
Precuneus	Other	8177	177	4.18	6	-56	26	
Left Superior Frontal Gyrus	Other	13721	297	4.06	-46	24	34	
AN-C	
Right Parietal	Self	13259	287	5.45	62	-24	34	
Left Insula and Inferior Frontal Gyrus	Self	12659	274	5.08	-38	0	-10	
Right Insula and Inferior Frontal Gyrus	Self	29938	648	5.00	42	12	-14	
Left Fusiform Gyrus	Self	9148	198	4.94	-38	-68	-10	
Right Fusiform Gyrus	Self	13490	292	4.46	34	-76	-10	
AN-WR	
Right Insula and Inferior Frontal Gyrus	Self	18157	393	5.43	38	12	-2	
Middle Cingulate	Self	13259	287	4.96	6	28	30	
Left Insula and Inferior Frontal Gyrus	Self	5359	116	4.93	-38	8	-6	
Precuneus	Other	7253	157	4.06	6	-60	30	
Right Temporoparietal Junction	Other	6237	135	4.02	42	-56	30	
	a Threshold was voxel P < 0.005 and cluster PFWE < 0.05 (extent of 101 voxels), using the F-contrasts for each group. Self or Other refers to whether the activation at this location were elevated in the F_Self condition relative to the F_Other condition (Self) or elevated in F_Other relative to F_Self (Other). Peak Z Score refers to the z-score at the MNI coordinates for the specified anatomical location. HC, healthy women, AN-C, currently-ill women with anorexia, AN-WR, weight-recovered women with anorexia.
b. T-contrast maps were used to create the corresponding figure, Figure S3, of the “Average Effect of Condition, All Subjects” to allow color-based displays of the Self and Other clusters. Table S8 reports on clusters based on the Z-score criteria. Slight differences may result (T-contrasts are more sensitive but less specific).	

Table S9. Current antidepressant use and â values of ROIs with Group Effects a 
	Med Use
(n = 19)b	No Med Use
(n = 21)b	Statistics	
	AN-C
(n = 12)	AN-WR
(n = 7)	AN-C
(n = 10)	AN-WR
(n = 11)	Group	Med	Interaction	
	Mean(SD)	Mean(SD)	Mean(SD)	Mean(SD)	F, p	F, p	F, p	
Social Identity-V2, Self - Friend	
          MPFC (-12, 48, 20)c	-0.39(0.9)	1.1(1)	0.41(0.5)	1.1(1.1)	11.9, 0.001d	1.9, 0.18	1.3, 0.27	
Social Identity-V2, Reflected - Self	
          L_IFG (-56, 8, 4)	-0.04(1.2)	-0.53(0.7)	-0.12(0.8)	-1.1(0.8)	5.6, 0.024e	1.2, 0.29	0.6. 0.43	
          R_IFG (36, 32, 8)	0.16(0.6)	-1.2(0.6)	-0.05(0.7)	-1.2(0.7)	32.6, <0.001	0.2, 0.67	0.3, 0.60	
          dACC (4, 32, 32)	0.71(1.0)	-1.5(1.6)	-0.74(0.7)	-2.4(1.8)	19.1, <0.001	7.0, 0.02	0.4, 0.56	
Social Identity-V2, Self-Agree – Self-Disagree	
           MPFC_AD (-12, 48, 20)	-0.31(0.4)	-0.33(0.4)	-0.29(0.5)	-0.15(0.4)	0.2, 0.68	0.5, 0.05	0.3, 0.56	
Faces, F_Self – F_Other	
           L_Fusi (-34, -60, -14)	0.58(0.4)	0.10(0.3)	0.53(0.5)	-0.32(0.4)	18.7, <0.001	2.2, 0.15	1.5, 0.24	
           R_Fusi (34, -76, -10)	0.55(0.4)	0.11(0.4)	0.73(0.4)	-0.43(0.7)	17.9, <0.001	0.9, 0.35	3.6, 0.07	
a.	All clusters identified in the whole-brain group ANOVAs (Table 2) were examined for medication effects by examining the extracted b-values in each region for both group effects and medication effects. AN-C, currently-ill women with anorexia, and AN-WR, weight-recovered women with anorexia. 
b.	For the faces task, there were only 18 AN-C that completed the task: 11 on antidepressants and 7 not. 
c.	The MNI coordinates for the Regions of Interest are indicated in parentheses in the following format (x, y, z). 
d.	Bold values in the statistics indicate significant results, using a adjusted significance threshold adjusted for the number of regions assessed (P = 0.007 (P = 0.05/7 ROIs). 
e.	Italics in the statistics indicate statistical trends (P < 0.05).


Figure S1. Social Identity-V2 task activations for all subjects: Self vs. Friend contrast
 
Overall main effect of condition for the Social Identity-V2 task, contrasting Self appraisal (red) with Friend appraisals (blue). Each T-contrast set to cluster PFWE < 0.05 (voxel P = 0.005, and cluster extent of 44 voxels). Note activations of the parietal regions in response to Self appraisals. Midline regions, including the MPFC and precuneus / posterior cingulate were more engaged during Friend appraisals. The corresponding table, Table S2, reports on the clusters identified in the Main Effect of Condition F-test. 


Figure S2. Social Identity-V2 task activations for all subjects: Reflected vs. Self contrast

Overall main effect of condition for the Social Identity-V2 task for Reflected (red) - Self (blue) appraisals. T-contrast set to cluster PFWE < 0.05 (voxel P = 0.005, and cluster extent of 41 voxels). Note activations of the temporoparietal junctions and precuneus more during Reflected Appraisals than Self Appraisals. The occipital lobe, fusiform gyri and cingulate are activated more for Self appraisals than Reflected Appraisals. The corresponding table, Table S6, reports on the clusters identified in the Main Effect of Condition F-test for Reflected – Self.

Figure S3. Faces task activations for all subjects:  F-Self vs. F-Other contrast


Overall main effect of condition for the Faces task, contrasting F-Self (red) with F-Other (blue). T-contrast set to cluster PFWE < 0.05 (voxel P = 0.005, with cluster extent of 101 voxels). Note strong activations of the middle cingulate, bilateral insula, bilateral parietal regions and inferior frontal gyri in response to F-Self images. More posterior regions, including the temporoparietal junctions and precuneus are engaged for F-Other images. The corresponding table, Table S8, reports on the clusters identified in the Main Effect of Condition F-test.


Figure S4.    Social Self Evaluation contrast is related to both eating disorder symptoms (A) as well as long-term recovery status (B). Note that the RMFG and dACC-EDEQ clusters in A partially overlap with the RIFG and dACC cluster in B. The LMFG in A is dorsal and did not overlap with the LIFG cluster in B. Peak coordinates, Z score, and extent of all clusters in Table 2 of manuscript. 

  A.	EDE-Q Regression, AN-C and AN-WR subjects.		B. Whole-Brain ANOVA: AN-C, AN-WR, and HC

Patient Perspectives S1

Social Identity-V2 Task

Self-Perception (Self – Friend): “The self-evaluations were really hard because I know I am not perfect and have more things to consider but everything good is true of my friend, and they must think good things of me, otherwise they wouldn't be my friend” – one AN-C subject

Self-Relevance (Self-Agree – Self-Disagree): “Disagreeing was hard because I had to make a decision about whether I am truly not like that whereas it is easy when I know I am like something” – one AN-WR subject

Social Evaluation (Reflected – Self): One AN-C subject reported “I did all of them correctly” after leaving the scanner. Since there are not right or wrong answers in the task, we asked for clarification. “I think I had all the same answers for each adjective, so if I agreed 'I believe I am something' then I also agreed 'My friend believes I am that same thing'.” Her neural data, akin to the rest of the AN-C group (Figure 2), showed minimal differences in neural regions engaged when comparing the process of evaluating her friends' perspective of herself (Reflected) and evaluating herself directly (Self). Of note, the instructions for the task included an example of different perspectives, such as “I might agree with the statement 'I believe I am frugal' but I would disagree with 'My husband believes I am frugal', because he and I have different ideas about what is worth spending money on.”. 

All of these comments suggest evaluations about oneself are challenging for women with and recovered from anorexia nervosa.

Faces Task: Headshots of subjects were obtained in a research office using standard lighting and rarely resulted in flattering images. All subjects were informed that the pictures were provided our apologies for the poor images, and told that we would destroy the images following the scan if so requested. Many of the AN-C subjects requested this, and several commented that viewing themselves was very hard, with one stated, “I was so relieved when the image was the other person and not me”. In contrast, many of the HC and the AN-WR subjects discussed how the stranger reminded them of someone they had known “She looked just like my college roommate, I could not stop thinking about her”, and rarely commented on their own images. These perceptions are consistent with the neural data showing increased activations in visual regions for the AN-C group when viewing their self-image relative to the stranger-image (Figure 3). 


Supplemental Analysis 1: Valence in the Social Identity V2 Task 

Effects of valence on self-related brain function were considered by reanalyzing data and ignoring the behavioral responses. There were insufficient stimuli were present to evaluate condition (self, friend, reflected), behavior (agree/disagree), and valence (positive/negative), as agree/disagree was confounded with valence in all subject groups. Consistent with the literature and our prior work, subjects typically agreed with few negative-valence terms and disagreed with few positive-valence terms. 

For this analysis, adjectives were divided into more positive terms (confident, considerate, curious, decisive, dependable, easygoing, friendly, honest, independent, intelligent, interesting, mature, optimistic, practical, quiet, realistic, reliable, sensitive, serious, sincere, truthful, tough, warm, wise) and more negative terms (clumsy, compulsive, conceited, critical, cynical, deceitful, dishonest, disrespectful, greedy, impractical, insecure, messy, moody, ordinary, phony, possessive, proud, reckless, sarcastic, selfish, spiteful, stubborn, timid, untruthful). The event-related design extracted the BOLD signal during the 4-s presentation of each statement and a general linear model created contrast images of each event (events: Social Identity-V2: Self-Positive, Self-Negative, Friend-Positive, Friend-Negative, Reflected-Positive, Reflected-Negative).  Our primary interest were overall differences related to positive and negative terms (Pos - Neg = (Self-Positive + Friend-Positive + Reflected-Positive) – (Self-Negative + Friend-Negative + Reflected-Negative), and self-specific positive and negative reflection (Self-Pos –Self-Neg). 

Importantly, although there were effects of condition meeting threshold for each ANOVA (Pos – Neg, 5 positive clusters; Self-Pos – Self-Neg, 9 clusters; threshold cluster PFWE < 0.05, voxel P < 0.005), there were no effects of group (AN-C, AN-WR, HC). These data suggest that valence alone does not account for the neural differences observed across the groups, and supports our interpretation that the differences related to the Self-Agree – Self-Disagree contrast are primarily related to the cognitive process of generating a self-relevant decision more than an effect of considering words of different valences. However, this is a post-hoc analysis, and we recognize that a different experimental design is required to fully assess how valence impacts the neural circuitry engaged for self-evaluation in anorexia nervosa.

Pos – Neg Clusters: 			Occipital (MNI -4, -84, 0; Z = 5.74; 1194 vox)
			 		Inferior Frontal Gyrus (MNI -40, 36, 0; Z = 4.80, 706 vox)
			 		Striatum/Thalamus/Midbrain (MNI 16, 16, 8; Z = 4.58, 299 vox) 
					 Inferior Frontal Gyrus (MNI 40, 24, 24; Z = 4.14, 108 vox)
					Right Precuneus (MNI 28, -60, 44; Z = 3.86, 69 vox)

Self-Pos – Self-Neg Clusters: 	Precuneus (MNI -12, -60, 16; Z = 6.23, 622 vox)
				     	Occipital (MNI -44, -72, 32; Z = 4.59, 112 vox)
				   	MPFC (MNI 0, 56, -4; Z = 4.44, 85 vox)
					Parahippocampal Gyrus (MNI 24, -24, -24; Z = 4.41; 88 vox)
			 	     	Middle Frontal Gyrus (MNI -20, 28, 44; Z = 4.30; 82 vox)
					Middle Temporal Gyrus (MNI 52, -64, 16; Z = 4.02; 50 vox)
				    	Middle Frontal Gyrus (MNI 44, -48, 12; Z = 3.99; 51 vox)
					Parahippocampal Gyrus (MNI -24, -40, -16; Z = 3.87; 61 vox)
				     	Occipital (MNI 40, -80, -8; Z = 3.71, 99 vox)


Supplemental Analysis 2: Weight and Symptom Effects 

The Social Identity-V2 ANOVAs were repeated for each contrast, excluding the six AN-C subjects that were in a normal weight range on the day of the scan and the six AN-WR subjects that reported high levels of symptoms (EDE-Q global > 3). The resulting comparisons included 16 AN-C, 12 AN-WR, and 19 HC. Because this post-hoc analysis removed about a third of the patient data, a less robust criterion of voxel P < 0.005 and a minimum cluster-size of 20 voxels was chosen.  Similar main effect of group clusters were observed in this smaller dataset. More clusters were observed in the Self-Friend contrast, although three of the clusters  (both MPFC and the dACC) were largely within the single cluster obtained in the larger analysis. Only one region, the left inferior frontal gyrus/insula (from the Reflected – Self contrast), was no longer observed after the removal of these twelve subjects. The additional regions obtained (Self-Friend, RIFG; Reflected – Self, caudate) were present in the original analysis if the criterion threshold was lowered to P < 0.005, 20 voxel extent. 

·	Self - Friend: 
a.	RIFG  (40, 28, 0) Z = 4.52, 36 vox
b.	MPFC (0, 60, 24) Z = 4.02, 84 vox
c.	MPFC (-8, 48, 0) Z = 3.96, 41 vox
d.	dACC (4, 32, 52) Z = 3.60, 23 vox

·	Self-Agree – Self-Disagree: 
a.	MPFC (-4, 47, -4) Z = 3.52, 31 voxels 

·	Reflected – Self: 
a.	dACC (4, 32, 28) Z = 3.86, 20 voxels
b.	Caudate (-16, 16, 8) Z = 3.80, 25 voxels
c.	RIFG (36, 28, 4) Z = 3.70, 51 voxels
